# Special Issue “Emerging Viruses in Aquaculture”

**DOI:** 10.3390/v13091777

**Published:** 2021-09-06

**Authors:** Manfred Weidmann, Mansour El-Matbouli, Weiwei Zeng, Sven M. Bergmann

**Affiliations:** 1 Institute of Microbiology and Virology, Medical School Brandenburg Theodor Fontane, 01968 Senftenberg, Germany; 2 Clinical Division of Fish Medicine, University of Veterinary Medicine, Veterinärplatz 1, 1210 Vienna, Austria; Mansour.El-Matbouli@vetmeduni.ac.at; 3 Guangdong Provincial Key Laboratory of Animal Molecular Design and Precise Breeding, School of Life Science and Engineering, Foshan University, Foshan 440605, China; zww8810303@163.com; 4 Institute of Infectology, Friedrich-Loffler-Institut (FLI), Federal Research Institute for Animal Health, Greifswald-Insel Riems, 17493 Greifswald, Germany; Sven.Bergmann@fli.de

According to the 2018 FAO report on aquaculture, there are 598 species of finfish, molluscs, crustaceans, and other organisms used in aquafarming around the world. Thus, the number of viruses potentially infecting these species is huge [1]. 

The overall economic impact due to virus infections in aquaculture can amount to a loss of up to 20% of biomass in a production cycle [2,3]. Aquaculture virology, therefore, remains a hot topic—a view apparently not shared by major institutions in the field. 

Aquatic viruses drift through the water passively, and their chance of hitting something in which to amplify is very low. Therefore, not surprisingly und unlike terrestrial viruses, aquatic viruses are very promiscuous, with a huge host range. Infectious pancreatic necrosis virus (IPNV), for example, is replicated in about 50 finfish species. A penned-in finfish shoal in a cage is an ideal situation for an aquatic virus. Once the infection establishes a hold, the basic reproduction number (R_0_) of the virus infection amongst the fish in the cage easily shoots beyond the threshold where only the host number can limit the R_0_.

The aim of this Special Issue was to collect peer-reviewed reports, perspectives, reviews, and research articles focusing on recent advances in the study of known and emerging aquaculture viruses.

The papers submitted reflect the diversity of aquaculture across freshwater and seawater. In keeping with the biggest output of 60% of total worldwide aquaculture production [1,4] a third of the papers present work from China presenting evidence on newly characterized viruses. Our call also attracted papers on WSSV, a major issue for shrimp production looking at local evolution in the wild and in aquaculture as well as some thorough work on shrimp miRNA responses to WSSV infection. A couple of papers are looking at aspects of the evolution of known (rhabdoviruses in trout, IPNV in salmon) and new viruses (reoviruses in carp, mimivirus in sturgeon) in finfish. We are convinced the assembled publications should provide interesting reading for the aquaculture virology community.
